# Ethnobotanical Survey of Culturally Important Plants and Mushrooms in North-Western Part of Croatia

**DOI:** 10.3390/plants13111566

**Published:** 2024-06-05

**Authors:** Ljiljana Krstin, Zorana Katanić, Katarina Benčić, Laura Lončar, Tanja Žuna Pfeiffer

**Affiliations:** Department of Biology, Josip Juraj Strossmayer University of Osijek, 31000 Osijek, Croatia; lkrstin@biologija.unios.hr (L.K.); zkatanic@biologija.unios.hr (Z.K.); katarinabencic23@gmail.com (K.B.); laura.loncar@gmail.com (L.L.)

**Keywords:** digestive ailment category, ethnomedicine, informant consensus factor, traditional knowledge, wild and cultivated plants

## Abstract

The Republic of Croatia is spread in geographical and climatic conditions that support a great diversity of habitats and associated plant taxa, many of which can be used for food or medicine. However, urbanization, loss of natural habitats, as well as changes in people’s dependence on the natural resources from the surrounding environment may lead to the loss of valuable knowledge about the use of plants and mushrooms. With the aim of studying and preserving this knowledge in the continental north-western part of Croatia, an ethnobotanical survey was undertaken at the two study areas—Valpovo and Đurđevac, which included a total of 17 settlements. A total of 103 informants, 65% female and 35% male, aged between 22 and 83 years, participated in an interview using pre-planned questionnaires. The informants reported 131 plants belonging to 55 families and 17 mushroom taxa. The largest number of plants belonged to the families of Rosaceae, Lamiaceae, Asteraceae, and Apiaceae. In both areas, the informants cultivate and also gather wild plants, but these practices are better preserved in the area of Đurđevac where 109 taxa from 47 families were recorded. In addition to cultivated and gathered plants, informants from the Valpovo area also reported the use of purchased plants. Plants and mushrooms are mostly used as food (21 plant taxa and 17 mushrooms), but plants also serve as medicine (68 taxa), as both food and medicine (35 taxa), feed for cattle (11 taxa), repellent (four taxa), and/or space freshener (two taxa). The most frequently used wild plants are *Chamomilla recutita*, *Mentha* x *piperita,* and *Urtica dioica,* while *Boletus edulis*, *Agaricus campestris,* and *Macrolepiota procera* are the most often used mushrooms. The results indicate that the local people in the studied north-western part of Croatia still nurture the practice of cultivating and gathering plants and that herbal remedies are considerably important among the informants. The study should be further extended to broaden and preserve valuable ethnobotanical knowledge and encourage the protection of culturally important plants of the studied area.

## 1. Introduction

Worldwide, all cultures have used plants for health promotion, disease prevention, and/or treatment since ancient times [1]. The use of herbal medicinal products and supplements has increased greatly over the past few decades with no less than 80% of the world population depending on them for their health care needs [2,3]. Of the estimated 250,000 flowering plants globally, between 50,000 and 70,000 are known to be used in traditional medicine [4]. The availability of medicinal plants, their low cost, and fewer side effects compared to standard medicines create a positive attitude towards traditional uses of plants [5,6,7]. Although modern science is focused on the determination of biological components in plants traditionally used for the treatment of various diseases, only 6% of globally known species have been screened for biological activity [8]. US Food and Drug Administration (FDA) has approved some drugs such as artemisinin (from *Artemisia annua*), quinine (from *Cinchona officinalis*), solamargine (from *Solanum dulcamara*), alitretinoin (from *Daucus carota*), and others that have been derived from natural sources [9]. Thus, ethnobotanical studies can enhance our understanding of traditional healthcare practices and provide valuable knowledge for new drug discovery.

Worldwide, ethnobotanical studies also provide insights into how local communities interact with natural resources such as wild edible plants. These plants are particularly consumed during times of food scarcity, poverty, extreme weather, or political unrest, and documentation of valuable indigenous knowledge of their use can contribute to conservation strategies [10,11,12]. Besides wild plants, some communities have a long tradition of using wild mushrooms as food, a source of income, and medicine [13]. Due to the high content of proteins, vitamins, minerals, fiber, antioxidants, and low content of cholesterol, mushrooms represent popular food [14] while their traditional uses for medicinal purposes lead to the discovery of alternative sources of synthetic medicines [15]. Thus, the preservation of valuable knowledge and the sustainable management of mushrooms can ensure the well-being of local communities.

Increasingly pronounced climate changes have a significant impact on plant species and are a serious threat to biological diversity and the disappearance of certain plants, especially those that are not adapted to high temperatures and long-term droughts. The effects of climate change could negatively affect the sustainability of medicinal plants in the researched areas and their availability to the local population. Therefore, there is a need to seriously address the protection of medicinal plants and their habitats through carefully planned protection strategies by the competent authorities [6]. The conservation of traditional knowledge is associated with at least seven sustainable development goals provided by Agenda 2030 [16], and ethnobotanical studies can significantly contribute to the conservation and sustainable use of natural flora. Ethnobotany connects local and scientific knowledge aiming to improve biological understanding for spatial conservation planning [17]. This linkage contributes to sustainability across the globe and plays an important role in assessments and policy formulation for biodiversity and ecosystem services [18].

Traditional practices in the use of wild plants depend on many factors including access to natural environments as well as the availability and accessibility of plant resources and the associated knowledge of their uses [19,20]. Due to its specific geographical and climatic conditions, the Republic of Croatia is rich in natural habitats [21] and has a great diversity of medicinal and aromatic plants [22]. Ethnobotanical studies carried out in the coastal or peri-coastal areas [23,24,25,26], in northern Dalmatia (Knin area) [27], Central Lika Region [28], and the continental part of Croatia [29] showed that some valuable practices have been preserved. However, in a large part of Croatia, ethnobotanical research has not been conducted and this valuable information which was not documented is in danger of being lost. Therefore, this study focused on the continental part of Croatia, settlements located in Koprivnica-Križevci County and Osijek-Baranja County, where the mosaic of habitats appears, thus maintaining high biodiversity [30]. The region of Koprivnica-Križevci County is mainly agricultural with cultivated fields and forests and a high number of vineyards. There are no pronounced industrial activities and traffic is relatively low in comparison with the urbanized part of Croatia [31]. Thus, in this area, rare and endangered species occur [32]. Osijek-Baranja County represents one of the most intensive areas of plant production among the counties in Croatia [33]. This flat area also has processing industries, forests, horticultural and protected areas, and plants [34]. To our knowledge, there is no database providing a list of plant taxa and mushrooms traditionally used for various purposes. However, the vicinity of the state borders, various infrastructure facilities and waterways of the Drava and Danube rivers, as well as the coexistence of different ethnic groups and population displacement are likely to have an impact on the preservation of these natural wealth, traditional ethnobotanical knowledge, and its application in both counties. The aim of this study was to document plant and mushroom species traditionally used for food, disease treatment, and/or health promotion by the local population. In addition to preserving valuable knowledge, detailed analysis of traditional practices can contribute to natural resource and biodiversity management, cultural preservation, and drug development.

## 2. Results

A total of 103 informants from the north-western part of Croatia participated in this research (67 females (65%) and 36 (35%) males), of which 43 (35 females and 8 males) were from the wider area of Đurđevac and 60 (32 females and 28 male) from the wider area around Valpovo. The age of the informants ranged from 22 to 83, with an average of 46.1 years, but the age structure of the informants from the two investigated areas differed. In the Đurđevac area, the largest number of informants were aged over 60 years (37.21%), followed by those aged 41 to 50 years (25.59%), and those aged 51 to 60 years (20.93%), while 16.28% were younger than 40 years old. In the Valpovo area, the majority of informants were aged between 22 and 40 years (61.67%), followed by those aged between 51 and 60 years (18.33%), and those aged between 41 and 50 years (11.67%), while the lowest number of informants were older than 60 years (8.33%). Most of the informants were secondary school graduates (53.46%) while others were university graduates (24.1%) and primary school graduates (21.1%). In addition to age, the informants in the two areas also differed in their level of education. Informants from the Valpovo area had a higher percentage of university-level education (36.6%) than informants from the Đurđevac area (11.6%).

In total, 131 plant taxa belonging to 55 families and 110 genera were identified, of which 55 taxa (5 purchased) were from 30 families in the Valpovo area, and 109 taxa from 47 families in the Đurđevac area (Table 1). One taxon belongs to Equisetidae, Ginkoidae, and Pinidae, respectively, and all others belong to Magnollidae. Considering the number of taxa belonging to each of the individual family, the families Rosaceae (19 taxa), Lamiaceae (13 taxa), Asteraceae (10 taxa) and Apiaceae (8 taxa) were the most diverse. Other families were represented with four or fewer taxa.

The degree of similarity between the plants recorded in the two investigated areas was determined using the Jaccard Index, which was 26.61%. Of the total number of taxa, 33 are common to both study areas (e.g., *Chamomilla recutita*, *Allium cepa*, *Allium sativum*, *Daucus carota*, *Mentha* x *piperita*, *Urtica dioica*, *Salvia officinalis*, *Origanum vulgare*, *Laurus nobilis*, *Calendula officinalis*, *Achillea millefolium*). Some informants use them for the same purposes, but sometimes their application differs (Table 1). For example, informants in both areas use *C. officinalis* for skin ailments such as wounds, scars, burns, stretch marks, and dry and infected skin, although informants from Đurđevac also use this species for the treatment of hemorrhoids and menstrual problems. Informants from both areas use *A. millefolium* for different purposes. In the area of Valpovo, it is used for various digestive (constipation, diarrhea, stomach pain) and menstrual problems, while in the Đurđevac area, it is used for urinary tract infection, dermatological problems, against inflammation, and as livestock feed. *Mentha* x *piperita* in the Valpovo area is used to treat sore throat, digestive problems, sinusitis, acne, and hormone imbalance symptoms, while in the Đurđevac area, it is used for neurological problems, detoxification, immune system, inhalation, and to maintain the cleanliness of the oral cavity.

A total of 22 plants (e.g., *Capsicum annum*, Piper nigrum, Olea europea, *Cinnamomum verum*, *Rubus plicatus*, *Ricinus communis*, *Papaver somniferum*, *Artemisia vulgaris*) are used only in the Valpovo area, while 76 taxa (e.g., *Hypericum perforatum*, *Tilia* sp., *Aronia* sp., *Equisetum arvense*, *Prunus cerasus*, *Prunus domestica*, *Sempervivum tectorum*, *Rubus caesius*, *Robinia pseudoacacia*) are used only in the Đurđevac area (Table 1).

**Figure 1:**
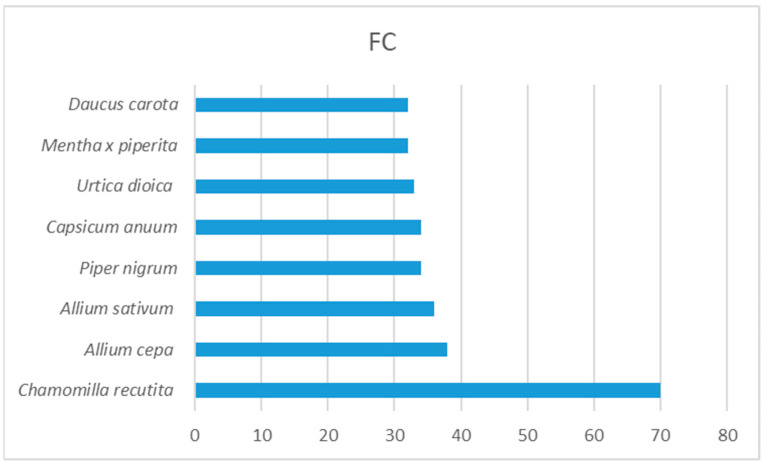
Plants with the highest frequency of citation (FC).

The relative importance of plant taxa was expressed by the RFC values which ranged from 0.01 to 0.68. The most commonly used plant with the highest RFC was *C. recutita* (0.68). Other taxa with high RFC were *A. cepa* (0.37), *A. sativum* (0.35), *P. nigrum* (0.33), *C. anuum* (0.33), *U. dioica* (0.32), and *Mentha* x *piperita* (0.31). The frequency of citations for *C. recutita* was 70, while this value was less than 40 for the other plants (Figure 1).

Some informants from the Valpovo area pointed out that they purchase certain plants (*Cinnamomum verum*, *Curcuma longa*, *P. nigrum*, *Syzygium aromaticum*, *Zingiber officinale*) and use them as spices, for tea, fresh or for medicinal purposes. Although these plants are not cultivated or gathered, the use of these plants is significant among the informants, especially *P. nigrum*. Additionally, the informants collect 74 cultivated plants and gather 52 wild plants. Most of the recorded plants are found in gardens, fields, or along the edges of forests.

Of the total number of plants, informants mostly used plant leaves (56 taxa), fruits (34 taxa), flowers (35 taxa), stems (19 taxa), roots (16 taxa), herbs (13 taxa), and seeds (10 taxa). Apart from these plant parts, the informants also use bulbs (*A. cepa*), whole plant (*Taraxacum officinale*), bark (*Ulmus minor*), twigs (*P. cerasus*), and corn silk (*Zea mays*).

The most commonly used herbal preparation is an infusion which is prepared from 67 plant taxa. Other preparations include juices (16 taxa), oils (15 taxa), creams (9 taxa), compresses (9 taxa), ointments (8 taxa), tinctures (8 taxa), macerates (6 taxa), syrups (5 taxa), liqueurs (4 taxa), wine (3 taxa), and schnapps (2 taxa) (Table 1).

Informants use plants most often for medicinal purposes (68 taxa) and as food (21 taxa), while 35 taxa are used for both food and medicinal purposes (e.g., *D. carota*, *Laurus nobilis*, *Origanum vulgare*, *Mentha* x *piperita*). However, plants are also used as livestock feed (11 taxa; *Cucurbita* sp., *U. dioica*, *Helianthus annuus*), for the preparation of alcoholic (*Juglans regia*, *Artemisia absinthium*) and non-alcoholic drinks (*Sambucus nigra*), for refreshing indoor spaces (*Lavandula angustifolia*) or as repellent against moths, mice, moles or mosquitoes (*Ricinus communis*).

From the plants used as food, the informants prepare salad (*T. officinale*), winter food (*Abelmoschus esculentus*), sauce (*Armoracia rusticana*), soup (*U. dioica*), jam (*Rosa canina*), fritters (*S. nigra*), cakes (*Castanea sativa*), but also use them as sweeteners (*Stevia rebaudiana*), spice (*Rosmarinus officinalis*), and honey (*R. pseudoacacia*).

**Figure 2:**
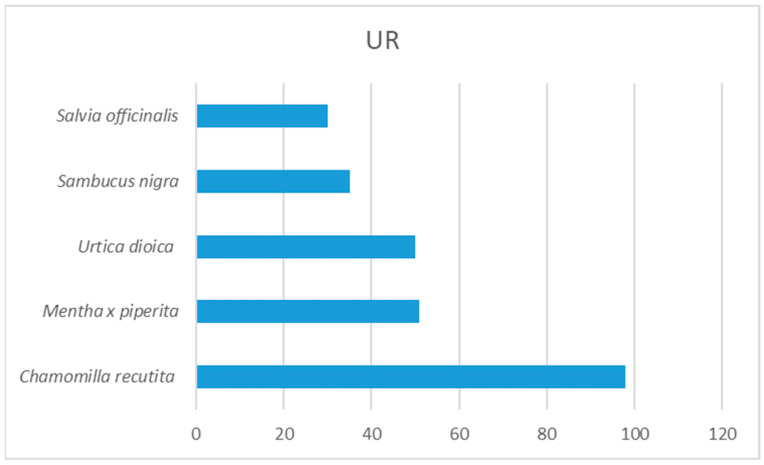
Plants with the highest value of use report (UR).

The plants used for medicinal purposes were classified into 14 categories, with a total of 851 disease use reports. Taxa with the highest use report are *C. recutita* (UR 98) reported by 70 informants, *Mentha* x *piperita* (UR 51) reported by 32 informants, *U. dioica* (UR 50), reported by 33 informants, and *S. nigra* (UR 35) reported by 22 informants (Figure 2).

The largest number of plants (45 taxa) are used to treat digestive problems, among which the most often reported are *Origanum vulgare* (UR 25) and *Mentha* x *piperita* (UR 25) (Table 2).

For the treatment of respiratory system disorders, 26 plant taxa are used, with *C. recutita* (UR 30) and *S. officinalis* (UR 20) being the most commonly mentioned plants. For the treatment of blood and immune mechanism disorders, *R. plicatus* (UR 11), *U. dioica* (UR 7), and *A. sativum* (UR 7) are the most popular plant among the 23 taxa within that category, while 23 taxa are successfully used to treat different dermatological diseases, and most often mentioned are *Calendula officinalis* (UR 23) and *C. recituta* (UR 19). A total of 20 taxa are effective in the treatment of urological diseases. Among them, *U. dioica* (UR 10) and *E. arvense* (UR 10) are the most effective (Table 2). Within the other categories, less than 20 taxa were recorded. *Sempervivum tectorum* is the only plant used to treat ear disorders.

Some plants were mentioned in more than one category. *Mentha* x *piperita* is used for the treatment of disorders belonging to the nine different categories, *C. recutita*, *U. dioica* and *M. officinalis* in eight and *T. officinale* in six categories.

In almost all categories, there were taxa that were mentioned only once, so the single-mentioned items index varied from 0.2 to 0.7.

The informant consensus factor (Fic) was calculated to determine the consensus of informants regarding the use of taxa in the treatment of certain categories of diseases. The informant consensus factor ranged from 0.22, for the treatment of general and unspecified diseases, to 1 for the treatment of ear diseases. Fic was high for eye disorders (0.9), followed by respiratory tract problems (0.84), dermatological system disorders (0.82), gynecological diseases (0.76), and digestive problems (0.76), while the low Fic value was calculated for muscle–skeletal disorders (0.47) (Table 2).

In addition to plants, in their diet, the informants also use a total of 17 mushrooms taxa belonging to seven families. According to the number of taxa used, the most represented are families of Boletaceae (five taxa), Agaricaceae (four taxa) and Russulaceae (three taxa). Three taxa were mentioned most frequently—*Boletus edulis*, *Agaricus campestris,* and *Macrolepiota procera* (73 UR), which were reported by 21 informants (Table 3). Mushrooms are used by informants to make soups and sauces, but some also fry them with eggs or dry them.

## 3. Discussion

In the studied north-western part of Croatia, cultivated and wild plants and mushrooms are often used as food, but plants also serve as medicine, feed for cattle, repellents, and/or space fresheners. For these various purposes, informants use more plant taxa (126 cultivated and gathered and 5 purchased plant taxa) than were reported in some previous studies conducted in the north-eastern (44 taxa) [29] and other parts of Croatia such as Central Lika Region (111 taxa) [28] or northern Dalmatia (123) [27]. Additionally, a lower number of traditionally used taxa was also reported in some other parts of the Balkan peninsula [35,36]. The most represented plants belong to Rosaceae (19 taxa), Lamiaceae (13 taxa), and Asteraceae (10 taxa) families, similar to other ethnobotanical studies conducted in the Balkan [37,38,39].

Although a lower number of informants were interviewed in the area of Đurđevac, a higher number of taxa and a higher number of those used only in this area were noted. These results may be related to the older, mainly female population included in the study. It was found that ethnobotanical knowledge increases with age and women report a higher number of culturally important plants [40]. Various taxa noted only in the Đurđevac area are also part of ethnobotanical practices in other countries. One of the most often used plants is *Hypericum perforatum*. It is a widely distributed and well-known plant used in traditional medicine for the treatment of depression, wounds and burns, diarrhea, pain, fevers, and other purposes [41]. It also represents an important plant in ethnobotany in the surrounding states such as Bosnia and Herzegovina [42], Serbia [43], or Italy [44]. *Prunus cerasus*, a natural hybrid between *Prunus avium* and *Prunus fruticose*, is known as a plant rich in various bioactive compounds [45]. Its ethnobotanical use as food or medicine was recorded in Spain [46], Kosovo [47], and Serbia [48]. *Tilia* spp. are widely distributed in Europe, and their traditional uses as medicine were noted in Bulgaria [49] and as a food in neighboring Hungary [50].

Informants in the Valpovo area use a lower number of plant taxa for food or medical purposes and they even buy certain plants in the store, which indicates the availability of new information and trends, not only preservation of traditional knowledge. This could also be related to the younger populations, the majority younger than 40 years old, included in the study who have access to different sources of knowledge. Also, informants with a university education, who are more numerous in Valpovo, probably stayed outside their place of residence during their education and thus had the opportunity to adopt new practices. For example, a high number of informants only in this area noted *Cinnamomum verum* as a plant for medicinal purposes. This plant naturally grows in several Asian countries, and due to its valuable biological compounds, it has been used in traditional medicine and has diverse culinary applications [51]. In addition, *Piper nigrum,* which is native to India and Malaysia, is rich in vitamins and minerals, has a high content of dietary fiber and various medicinal properties, and is used in traditional medicine [52]. Thus, allochthonous species bought in markets could also be important for local people [53]. These results point to important aspects for future ethnobotanical surveys which, in addition to the practices of the informants, should thoroughly investigate the source of knowledge, the informants’ habit of using the available literature or the internet as a source of information on the benefits of plant use. It could also be very informative to encourage them to remember and compare their practices with those of older family members.

This study also showed that plant cultivation represents an important practice, particularly in the area of Đurđevac. Some inhabitants in both study areas produce and collect different plants in their own gardens and use them for various purposes. Worldwide, home gardens are an important source of food and ensure food security through access, availability, use, and sustainability [54,55]. Their structure and diversity depend on socioeconomic factors, cultural values, and the interests of the owners [56]. One of the species most frequently cultivated by the informants and used as food and for medicinal purposes is *Allium cepa*, a bulbous perennial or biennial monocot plant. It possesses anti-cancer, anti-diabetic, and anti-platelet properties; it represents important traditionally used plants for the treatment of cough, asthma, inflammatory disorders, dysentery, ulcer, wounds, and other disorders [57,58]. Cultivated *Capsicum annuum* generally contains a variety of essential nutrients and bioactive compounds displaying antioxidant, antimicrobial, antiviral, anti-inflammatory, and anticancer activity [59], while *Daucus carota* has cytotoxic, antioxidant, antidiabetic, antimicrobial, hypotensive, gastro-protective, nephro-protective, hepato-protective, cardioprotective, and many other effects and is thus often used not only as food but also as a cure in traditional medicine [60,61].

Worldwide, traditional gathering and uses of wild plants declines due to the increased accessibility of market food products [62], the erosion of traditional knowledge and skills needed for the successful determination of edible taxa and their uses [63], as well as due to the generally accepted opinion that this practice is old-fashioned and a symbol of poverty [64]. However, in many local communities, these practices represent important food sources, and in modern societies, they become more popular as a result of the increasingly frequent use of wild plants in local gastronomy as well as due to their potential as a secure and sustainable source of food and dietary elements [65]. The gathering of wild plants in the studied areas also contributes to nutrition and healing, although there are some differences in the plant taxa used by the informants. This could be associated with differences in traditional knowledge and practices of various ethnic groups that inhabit Valpovo (mostly Croatians) and Đurđevac (e.g., Croatians, Germans, Hungarians, Serbians) areas. They probably retain some traditional practices but also exchange their valuable knowledge, leading to the homogenization of local plant utilization [66].

The most commonly used wild plant in the studied areas is *Chamomilla recutita*. The use of chamomilla is also recorded in other ethnobotanical studies in Croatia [28,29]. This species is widely distributed and used in traditional medicine for the treatment of infections, respiratory, gastrointestinal, and liver disorders as well as a sedative, antispasmodic, antiseptic, and antiemetic [67]. Besides chamomilla, *Mentha* x *piperita* is also frequently used as medicine. Widely distributed Mentha species have diverse applications recorded by numerous ethnobotanical studies [42,68,69]. They possess antimicrobial, antidiabetic, cytotoxic, and antioxidant potential, they are effective in curing cardiovascular diseases, and could be used as herbal medicine directly or indirectly as food ingredients [70]. As in this study, widely traditionally used as medicine are also *Urtica dioica*, *Melisa officinalis,* and *Taraxacum officinale*, which possess diverse bioactive constituents useful in the prevention and treatment of various disorders [71,72,73].

Leaves, fruits, and flowers are the most used plant parts in remedy preparation in both areas. Due to the high amount of bioactive compounds and their easy collection and preparation, leaves are more frequently used parts of plants in various ethnomedicinal studies [69,74].

Apart from wild plants, informants, mostly women, collect mushrooms in both study areas. This tradition, which usually depends on the available habitats suitable for mushrooms growth, has also been reported in other parts of Croatia [28,75]. Since there are a variety of habitats in the surroundings of Valpovo and Đurđevac such as abandoned arable lands, forests, meadows, and grasslands, it is not surprising that informants state that they use as many as 17 mushroom taxa in their diet. Generally, mushrooms are collected for food in many countries all over the world [13,64,76], often representing an important source of income for the local rural inhabitants [14]. The informants most often collect *Boletus edulis*. This mushroom, harvested in summer, is rich in polysaccharides, alkaloids, and other valuable compounds [77]. Thus, it represents valuable species also collected in neighboring countries [78,79,80]. Mushroom *Agaricus campestris* is easily recognizable and commonly occurs in hills and meadows, urban and peri-urban habitats [81], and it is widely used in ethnomedicine [79,80,82]. Also often collected is *Macrolepiota procera*. This mushroom contains diverse bioactive components, minerals (particularly K, Mg, and Se), and dietary fiber, and it has potential therapeutic and pharmacological anticancer, anti-inflammatory, immunomodulatory, antioxidant, and antimicrobial properties [83]. Its traditional uses are also widely recorded [79,80].

## 4. Materials and Methods

### 4.1. Study Sites

The study was conducted in the settlements of two cities located in the north-western part of Croatia—Valpovo and Đurđevac.

The town of Valpovo located near the Croatian-Hungarian border belongs to the Osijek-Baranja County. It lies in the typical accumulation plain formed by river flows. The area has a uniform and young relief, characterized by a slight unevenness of the terrain from 87 to 101 m altitude. Three types of relief can be observed: terraced lowlands, flood plains, and fluvial swamps. Valpovo has a warm moderately rainy climate with extremely continental features characterized by large annual temperature fluctuations and distribution of rainfall. The town area comprises eight settlements: Valpovo, Harkanovci, Ivanovci, Ladimirevci, Marjančaci, Nard, Šag, and Zelčin. These are the most ecologically valuable natural areas rich in rare and endangered species. The settlements of Valpovo, Harkanovci, Nard, and Šag, located on the right bank of the Drava River, belong to the protected Mura-Drava Regional Park and the NATURA 2000 network areas, while the wider area of Valpovština is included in the Mura-Drava-Danube Transboundary Biosphere Reserve [84].

The town of Đurđevac belongs to the Koprivnica-Križevac County. It is only 10 km from the state border with Hungary and approximately 100 km from the border with Slovenia. The relief of the area includes pleistocene terraces, alluvial plain, Sands of Đurđevac, and the Bilogora hill. The climate of the area changes from the moderately semi-humid to the steppe–arid Pannonian climate zone. There are many watercourses, artificial lakes, ponds, and wet habitats. Only 10 km from Đurđevac, the Drava River flows have a significant influence on groundwater level. A large part of the area is covered with forests. Due to the intensive anthropogenic influences, the original plant cover disappeared, but meadows, pastures, vineyards, and orchards have developed. There are the Sands of Đurđevac, a special geographical and botanical reserve characterized by unique and diverse flora, as well as Forest Park Borik. The town area comprises nine settlements [85].

Both Valpovo and Đurđevac areas are located along important roads and have developed agriculture and tourism.

### 4.2. Data Collection

**Figure 3:**
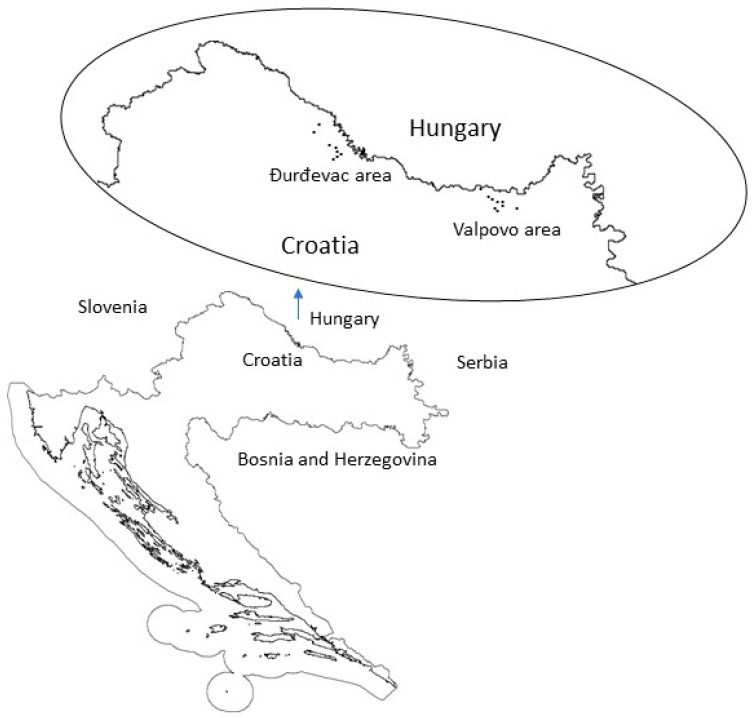
Location of the study area in the north-western part of Croatia.

The study was conducted from 2019 to 2022 in a total of 17 settlements in Valpovo (Valpovo, Gat, Veliškovci, Črnkovci, Donji Miholjac, Podravski Podgajci, Sveti Đurađ, Marijanci, Kunišinci) and Đurđevac area (Đurđevac, Budančevica, Kalinovac, Kloštar Podravski, Molve, Prugovac, Suha Katalena, Virje) (Figure 3). The study was carried out following the International Society of Ethnobiology Code of Ethics [86]. The interviews were conducted through pre-planned questionnaires (Table S1). Local people who declared that they use medicinal plants were asked to participate in the study voluntarily. All participants were informed of the purpose of the interview and how the data collected would be used, and all provided their informed consent before the interview continued. To ensure confidentiality and protect the rights of the informants, questionnaires were not associated with the name and surname or any other data that could be used for the identification of the informants. Completed questionnaires were available only to the authors of the study. The questionnaires were conducted orally with all respondents. Two co-authors of the paper come from the studied areas, and they helped establish communication with the informants. Informants were selected using the snowball sampling approach without any selection based on sex, age, or social status. The language used in the interviews was Croatian. The informants were first asked to provide general socioeconomic information such as gender, age, residency location, and level of education. Afterward, the informants were asked to list the plants that they use with a focus on plant names, parts of the plant that usually were collected, how they were processed and for what purposes, and also to list the mushrooms they use. The interviews were performed face to face in the informant’s home and/or in the field, allowing for informants to recognize and show individual plants from nature. The plant taxa were mostly determined in the field using the standard keys [87,88,89]. The taxa which could not be determined in the field were collected for additional verification or determination in the laboratory using a stereo microscope or a light microscope. Scientific names and author citations of identified plant taxa were checked using the Flora Croatica Database [88]. The dried plants were herbarized following standard protocols and stored at the Department of Biology University of Josip Juraj Strossmayer in Osijek.

### 4.3. Data Analysis

The ethnobotanical data obtained from the informants were entered in Microsoft Excel and analyzed quantitatively using the frequency of citation (FC), the relative frequency of citation (RFC), the number of use report (UR), the Jaccard Index (JI), the single-mentioned item index (SM), and the informant consensus factor (Fic).

To determine the local importance of each taxa, the relative frequency of citation (RFC) was calculated. The RFC value indicates the consensus among informants on reported taxa, and it is calculated using the following formula:

(RFC = FC/N),(1)

where FC is the number of informants who mentioned the use of a plant species and N is the total number of informants participating in the survey [90]. The value of the RFC index varies from 0, when nobody mentions the plant taxa as useful, to 1, when all informants mention the plant taxa as useful. FC, together with UR (provides information on the use of a plant or a plant part used by one informant for a specific medical category multiplied by the number of informants mentioning such use), is mainly used to determine the accuracy of ethnobotanical data. A higher value of UR points to a higher level of agreement among informants [91].

The Jaccard Index was used to compare the recorded plant taxa used in both researched areas. The formula to calculate JI index was

JI(%) = (C/(A + B − C)) × 100,(2)

where A is the number of taxa in sample A, B is the number of taxa in sample B, and C is the number of taxa common to A and B [92].

Concerning medicinal properties and application for medicinal purposes, the reported plant taxa were grouped into fourteen ailment categories: A—general and unspecified, D—digestive, B—blood, blood-forming organs, and immune mechanisms, T—endocrine/metabolic and nutritional, P—psychological, N—neurological, F—eye, H—ear, K—cardiovascular, R—respiratory, S—skin, L—musculoskeletal, U—urological, and X—female genitals.

A single-mentioned item index (SM) for each ailment category was calculated by the following formula:

SM = ntr/nt,(3)

where ntr is the number of taxa that are reported in each category only once and nt is the number of taxa used in each category [93]. The higher the SM value, the more single-mentioned plant taxa were included in the ailment category and the higher the disagreement between informants.

Following [94], the informant consensus factor (Fic) was calculated as follows:

Fic = (nur − ntaxa)/(nur − 1),(4)

where nur = number of use reports in each category and nt = number of taxa used in each category. The Fic factor ranges from 0 to 1. A higher value of the factor indicates that a large number of informants mention a relatively small number of plants, while a lower value indicates that the informants do not agree about the plant taxa used in the treatment within a category of ailment [94].

## 5. Conclusions

The study showed that informants in the north-western part of Croatia still nurture the practice of cultivation and gathering of plants for nutritional and medical purposes. Also, the collecting of various mushrooms taxa still represents an important food source. Both traditional practices offer economic and ecological benefits to informants and suggest that gathering could be related to the availability of diverse preserved habitats, rich in culturally important plants. Thus, further extended studies should be conducted to broaden and preserve our knowledge of plant and mushroom applications in the north-western part of Croatia. Due to a lack of previous investigation, the obtained data cannot be used to provide a better understanding of trends in plant and mushrooms applications or changes in practice over the years, which somewhat limits this research. However, this study provided documented data from previously unexplored areas, and therefore it could serve as the basis for the initiation of activities focused on plant protection by the competent authorities. It is particularly important to ensure continuous monitoring of plant and mushroom taxa in order to react in a timely manner. Furthermore, the results obtained contributed to raising awareness and promoting plants and their uses, which should be continued through systematic education of both young and elderly people, workshops, citizen science projects, and other forms of promotion.

## Figures and Tables

**Table 1:** List of plants used in the north-western part of Croatia.

Botanical Name	Family	Local Name	Part Used	Preparation	Uses	FC	Status
*Abelmoschus esculentus* (L.) Moench *	Malvaceae	bamija	leaf, fruit	spice, winter stores	food, T: diabetes	1	c
*Achillea millefolium* L. **	Asteraceae	stolisnik	flower, leaf	infusion, fresh, ointment, macerate, tincture	D: digestive problems, constipation, diarrhea, stomach pains; X: menstrual problems; L: inflammation; U: urinary tract infection; S: dermatological problems; animal food	23	w
*Aesculus hippocastanum* L. **	Hippocastanaceae	divlji kesten	fruit	ointment, tincture	L: back and shoulder pain; K: varicose veins	8	w
*Alchemilla vulgaris* L. **	Rosaceae	vrkuta	flower, leaf	infusion	D: digestive problems; X: polycystic ovaries; T: hormone imbalance	4	c
*Allium cepa* L. **	Amaryllidaceae	crveni luk	bulb, onion peel	spice, fresh, infusion	R: respiratory problems; K: cardiovascular; food	38	c
*Allium sativum* L. **	Amaryllidaceae	češnjak	bulb, onion peel	raw, chopping, spice	K: hypertension; B: immune system booster; R: otitis; food	36	c
*Allium schoenoprasum* L. +	Amaryllidaceae	vlasac	leaf	spice	food	2	c
*Allium ursinum* L. *	Amaryllidaceae	medvjeđi luk	leaf	fresh	food	1	w
*Althaea officinalis* L. +	Malvaceae	bijeli sljez	root, flower	infusion	R: cough, expectoration	4	w
*Aloe vera* L. *	Asparagaceae	aloja	leaf	cream, juice	B: immune system; S: skin redness	1	c
*Anethum graveolens* L. +	Apiaceae	kopar	leaf	spice	food	3	c
*Apium graveolens* L. **	Apiaceae	celer	leaf, root	fresh juice, infusion	U: urinary tract infection, detoxification; T: weight loss; X: menstrual irregularity; K: cholesterol	20	c
*Arctostaphylos uva ursi* (L.) Spreng. *	Ericaceae	medvjetka	leaf	infusion	U: urinary tract infection	1	c
*Armoracia rusticana* P. Gaertn., B. Mey. et Scherb. **	Brassicaceae	hren	root	decoction, inhalation, spice, sauce	R: sinusitis; L: musculoskeletal; food	5	w
*Aronia* sp.*	Rosaceae	aronija	fruit, leaf	jam, infusion, juice, wine, fresh	K: cardiovascular; B: for better blood count; D: digestive problems; food	10	c
*Artemisia absinthium* L. *	Asteraceae	gorski pelin	leaf, herb	infusion	D: gastrointestinal problems	4	w
*Artemisia vulgaris* L. +	Asteraceae	obični pelin	leaf	infusion	D: nausea, stomach pain, digestive parasites	6	w
*Avena sativa* L.*	Poaceae	zob	seed	infusion	D: gastrointestinal problems; L: musculoskeletal; U: urinary tract infection	2	c
*Beta vulgaris var. conditiva* L. +	Chenopodiaceae	cikla	root	pickled, fresh juice	B: anemia; food	2	c
*Betula pendula* Roth **	Betulaceae	bijela breza	leaf, buds	infusion	U: urinary tract infection; R sore throat	2	w
*Brassica oleracea* L. **	Brassicaceae	obični kupus	leaf	compression	A: fever; L: joint pain	4	c
*Calendula officinalis* L. **	Asteraceae	ljekoviti neven	flower, leaf	ointment, infusion, macerate, cream, tincture	S: wounds, scars, burns, stretch marks, dry skin, infection; K: hemorrhoids, varicose veins; X: menstrual problems	24	c
*Cannabis sativa* L. +	Cannabaceae	sjetvena konoplja	flower	inhalation	P: agitation; L: pain	2	c
*Capsicum anuum* L. +	Solanaceae	paprika	fruit	spice	food	34	c
*Carum carvi* L. **	Apiaceae	kim, kumin	seed	spice, infusion, oil	D: gastrointestinal problems; animal food; food	5	c
*Castanea sativa* L. *	Fagaceae	pitomi kesten	fruit	boiling	food	1	c
*Centaurium erythraea* Rafn *	Gentianaceae	štitasta kičica	herb	infusion	D: gastrointestinal problems	1	w
*Ceratonia siliqua* L. *	Fabaceae	rogač	fruit	chopping	D: gastrointestinal problems; R: allergies; food	1	c
*Chamomilla recutita* (L.) Rauschert **	Asteraceae	prava kamilica	flower	infusion, compression, inhalation, cream, ointment	R: sore throat, respiratory problems, cold; F: eye inflammation; S: inflammatory skin reaction, infection; P: insomnia, relaxation, agitation; X: menstrual problem; D: gastrointestinal problems, diarrhea; N: neurological; F: eye inflammation; A: fever	70	w
*Chelidonium majus* L. *	Papaveraceae	rosopas	herb, flower	fresh, ointment	S: dermatological problems	3	w
*Cinnamomum verum* J. Presl +	Lauraceae	cimet	bark	spice	T: blood sugar level balancing	14	p
*Citrullus lanatus* (Thunb.) Matsum. et Nakai *	Cucurbitaceae	lubenica	seed	infusion	U: urinary tract infection	1	c
*Citrus limon* (L.) Burm. f. *	Rutaceae	limun	fruit	limoncello	food–drink	1	c
*Coriandrum sativum* L. +	Apiaceae	sjetveni korijander	leaf	spice	food	1	c
*Cornus mas* L. *	Cornaceae	drijen	fruit	tincture, jam, fresh	D: gastrointestinal problems; food	2	w
*Crataegus monogyna* Jackq. +	Rosaceae	bijeli glog	flower	infusion	K: strengthening the heart, hypertension	1	w
*Cucurbita* sp. *	Cucurbitaceae	bundeva	fruit, seed	oil	animal food	1	c
*Curcuma longa* L. +	Zingiberaceae	kurkuma	root	spice	food	5	p
*Cydonia oblonga* Mill. **	Rosaceae	dunja	leaf, fruit	infusion, fresh	D: gastrointestinal problems; food; space freshener	4	c
*Cymbopogon citratus* D.C. Stapf +	Poaceae	limunska trava	stem, leaf	infusion	P: insomnia	2	c
*Daucus carota* L. **	Apiaceae	mrkva	root	fresh juice, maceration, salad, soup	S: oil for sun; F: eyesight; B: immune system; T: lose weight, vitamin A; food	32	c
*Epilobium parviflorum* Schreb *	Onagraceae	vrbovica	leaf, herb	infusion	U: urinary tract infection	1	w
*Equisetum arvense* L. *	Equisetaceae	poljska preslica	herb	infusion	U: urinary tract infection; D: gastritis	11	w
*Ficus carica* L. *	Moraceae	smokva	fruit, leaf	fresh, jam, cream	S: dermatological problems, infection, viral warts; food	3	c
*Foeniculum vulgare* Mill. *	Apiaceae	komorač	leaf, seed, root	infusion	D: gastrointestinal problems; U: detoxification	1	c
*Fragaria vesca* L. *	Rosaceae	šumska jagoda	fruit	fresh	food	1	w
*Galium mollugo* L. *	Rubiaceae	livadna broćika	herb	infusion	U: detoxification	1	w
*Galium verum* L. **	Rubiaceae	prava broćika	herb	infusion	R: respiratory problems; B: immune system; U: detoxification, urinary tract problems	3	w
*Ginkgo biloba* L. +	Ginkgoaceae	ginko	leaf	infusion	N: concentration, memory	3	c
*Helianthus annuus* L. *	Asteraceae	suncokret	seed	fresh, dry	animal food	1	c
*Helichrysum italicum* (Roth) G Don **	Asteraceae	smilje	flower, leaf	cream, infusion, oil	S: skin care; R: sore throat; P: anxiety	3	c
*Helleborus niger* L. *	Ranunculaceae	crni kukurijek	root	fresh	animal food	1	w
*Hypericum perforatum* L. *	Clusiaceae	gospina trava	flower	cantarion oil, infusion, cream	N: neurological problems; S: dermatological problems, infection, against insect bites, sunburn; P: antidepressant, calming down	12	w
*Juglans regia* L. **	Juglandaceae	pitomi orah	leaf, fruit	maceration, oil, liqueur, fresh, cake	L: rheumatism; D: gastrointestinal problems, stomach problems; S: hair loss, dermatological problems; N: memory; food	9	c
*Lamium purpureum* L. *	Lamiaceae	crvena mrtva kopriva	flower	honey	food	1	w
*Laurus nobilis* L. **	Lauraceae	obični lovor	leaf	spice, infusion, ointment	R: respiratory problems; K: cardiovascular; food	24	c
*Lavandula angustifolia* Mill. **	Lamiaceae	uskolisna lavanda	flower	oil, infusion, repellent, dry flowers, cream	S: stretch marks, burns, scars, dry skin, disinfection; N: neurological problems, headache; P: relaxation, insomnia; space freshener; repellent	13	c
*Lavandula* sp. *	Lamiaceae	lavandin	flower	oil, hydrolat	N: neurological problems	1	c
*Levisticum officinale* W.D.J. Koch *	Apiaceae	ljekoviti ljupčac	leaf	spice	food	1	c
*Lilium candidum* L. *	Liliaceae	bijeli ljiljan	flower, root	compression, tincture	N: neurological problems, S: dermatological problems	1	c
*Linum usitatissimum* L. *	Linaceae	lan	seed	mucus	animal food	1	c
*Lycium barbarum* L. *	Solanaceae	obični vučac	fruit	fresh, jam	food	1	c
*Malus pumila* Mill. *	Rosaceae	jabuka	fruit	compression, infusion	D: gastrointestinal problems; food	3	c
*Malva sylvestris* L. *	Malvaceae	šumski sljez	flower, root	infusion	R: respiratory problems, cold	6	w
*Melissa officinalis* L. **	Lamiaceae	ljekoviti matičnjak	flower, leaf	infusion, syrup	P: agitation, anxiety, depression, calming down; D: stomach ache, cleanliness of the oral cavity; N: neurological problems; B: immune system; A: analgesic; U: detoxification; R: for inhalation; K: tachycardia; food	12	c
*Mentha* x *piperita* L. **	Lamiaceae	paprena metvica	leaf, herb	inhalation, infusion, tincture, syrup, spice, fresh, liqueur	T: hormone imbalance; R: sore throat, for inhalation, sinusitis; D: digestive problems, cleanliness of the oral cavity, cramps; S: acne; N: neurological problems; B: immune system; A: analgesic; U: detoxification; P: calming down; food	32	c
*Mentha* sp. *	Lamiaceae	menta	leaf	infusion	D: gastrointestinal problems	1	w
*Mespilus germanica* L. *	Rosaceae	mušmula	fruit	fresh	B: immune system; food, animal food	2	c
*Morus alba* L. *	Moraceae	bijeli dud	leaf	infusion	D: gastrointestinal problems; K: cardiovascular	1	c
*Morus nigra* L. *	Moraceae	crni dud	fruit	fresh	D: gastrointestinal problems; food	1	c
*Nigella sativa* L. +	Ranunculaceae	crni kim	seed	oil	D: digestive problems	2	c
*Ocimum basilicum* L. **	Lamiaceae	bosiljak	leaf	spice	U: kidney disease; food	13	c
*Olea europea* L. +	Oleaceae	maslina	fruit	oil, raw	S: dry skin, burns, sun protection; D: constipation	15	c
*Origanum majorana* L. *	Lamiaceae	vrtni mažuran	leaf, flower	spice	food	1	c
*Origanum vulgare* L. **	Lamiaceae	obični mravinac	leaf	oil, spice	D: digestive problems, gastritis; food	27	c
*Papaver somniferum* L. +	Papaveraceae	pitomi mak	flower, seed	spice	food	9	c
*Petasites hybridus* (L.) P. Gaertn., B. Mey. et Schreb. *	Asteraceae	obični lopuh	root	fresh	animal food	1	w
*Petroselinum crispum* (Mill.) A. W. Hill *	Apiaceae	peršin	herb	infusion, smoothie, fresh	U: urinary tract infection, detoxification, B: immune system; D: digestive	2	c
*Pinus sylvestris* L. *	Pinaceae	bor	needles	infusion, syrup	R: respiratory problems; B: immune system	1	w
*Piper nigrum* L. +	Piperaceae	crni papar	fruit	spice	food	34	p
*Plantago lanceolata* L. *	Plantaginaceae	uskolisni trputac	leaf	compression, infusion	R: respiratory problems; S: bleeding, infection	4	w
*Plantago major* L. **	Plantaginaceae	veliki trputac	leaf, root	syrup, compression, infusion	R: sore throat, respiratory problems; S: bleeding, infection	7	w
*Potentilla anserina* L. *	Rosaceae	guščarski petoprst	leaf	fresh	B: anemia	1	w
*Prunus armeniaca* L. *	Rosaceae	marelica	fruit	jam	D: for digestion; food	3	c
*Prunus cerasus* L. *	Rosaceae	višnja	fruit, twigs	liqueur, fresh, infusion, juice, pie filling, jam	D: gastrointestinal problems; A: analgesic; food	7	c
*Prunus domestica* L. *	Rosaceae	šljiva	fruit	schnapps, jam, infusion	D: gastrointestinal problems; food	5	c
*Prunus dulcis* (Mill.) D.A.Webb *	Rosaceae	badem	fruit	oil	S: skin care; food	1	c
*Prunus spinosa* L. *	Rosaceae	trnina	flower, fruit	infusion, jam	D: gastrointestinal problems; K: cardiovascular	1	w
Pulmonaria officinalis L. *	Boraginaceae	ljekoviti plućnjak	leaf	infusion	R: respiratory problems	1	w
*Pyrus* sp. *	Rosaceae	kruška	fruit	fresh, schnapps	D: gastrointestinal problems	2	c
*Quercus* sp. *	Fagaceae	hrast	bark	infusion	D: gastrointestinal problems, gastritis; food for animals	2	w
*Ribes nigrum* L. *	Grossulariaceae	crni ribiz	leaf	infusion	D: gastrointestinal problems; K: cardiovascular	1	c
*Ribes rubrum* L. *	Grossulariaceae	crveni ribiz	leaf, fruit	infusion, wine, fresh, jam	B: immune system; U: detoxification; food	2	c
*Ribes uva-crispa* L. *	Grossulariaceae	ogrozd, trnoviti ribiz	fruit	fresh	food	1	c
*Ricinus communis* L. +	Euphorbiaceae	obični ricinus	seed, whole plant	oil, fresh plant	S: allergy, dry skin, acne; K: hemorrhoids; repellents for mice, moles and mosquitoes	7	c
*Robinia pseudoacacia* L. *	Fabaceae	bagrem	flower	honey, infusion, fresh	R: respiratory problems, cold; B: immune system; food	5	w
*Rosa canina* L. **	Rosaceae	pasja ruža	fruit	infusion, jam, raw fruit, fresh juice	D: gastrointestinal problems; U anti-infective; R: sore throat, influenza; B: immune system	17	w
*Rosmarinus officinalis* L. **	Lamiaceae	ružmarin	herb, leaf	spice, maceration, fresh	N: for brain; K: for circulation; food	18	c
*Rubus caesius* L.*	Rosaceae	modrosiva kupina	fruit, leaf	wine, juice, infusion, fresh, jam	K: cardiovascular; B: immune system; food	5	w
*Rubus idaeus* L. *	Rosaceae	malina	fruit, leaf	jam, juice, infusion, fresh	B: immune system; food	3	c
*Rubus plicatus* Weihe et Nees +	Rosaceae	nabrana kupina	fruit	fresh, wine	B: anemia	11	c
*Rumex acetosa* L. *	Polygonaceae	kiselica	leaf	fresh	food	1	w
*Rumex* sp. *	Polygonaceae	štavelj	flower	infusion	D: gastrointestinal problems	1	w
*Ruta graveolens* L. *	Rutaceae	ruta	herb	dry, tincture	repellent	1	c
*Salix alba* L. *	Salicaceae	bijela vrba	bark	infusion, compression	B: anemia; A: analgesic; food animal	1	w
*Salvia officinalis* L. **	Lamiaceae	ljekovita kadulja	leaf, flower	infusion, gargling, liqueur, juice	R: sore throat, cough, angina; D: gastrointestinal problems; R: respiratory problems; X: menstrual problems; S: anti-infective	29	c
Sambucus nigra L. **	Caprifoliaceae	crna bazga	leaf, flower, fruit	juice, infusion, jam, elderflower fritters	R: respiratory problems, allergies, cold, influenza; B: immune system booster; D: gastrointestinal problems	22	w
*Satureja hortensis* L. *	Lamiaceae	vrtni čubar	leaf, flower	spice	D: gastrointestinal problems; food	1	c
*Sempervivum tectorum* L. *	Crassulaceae	čuvarkuća	herb, leaf	fresh juice	H: earaches; S: after an insect bite, used to remove corns	6	c
*Solanum tuberosum* L. *	Solanaceae	krumpir	fresh tuber	compression	A: body temperature, fever	1	c
*Sorbus domestica* L. *	Rosaceae	oskoruša	fruit	fresh, liqueur	D: gastrointestinal problems	1	w
*Stevia rebaudiana* Bertoni **	Asteraceae	slatka stevija	leaf	sweetener, spice	food	2	c
*Symphytum officinale* L. *	Boraginaceae	gavez	whole plant, root	infusion, tincture, ointment, cream	D: gastrointestinal problems; R: respiratory problems; L: twist, swelling, sprain	4	w
*Syzygium aromaticum* (L.) Merr L.M. Perry +	Myrtaceae	klinčić	buds	infusion	D: stomach ache	1	p
*Tanacetum vulgare* L. *	Asteraceae	obični vratić	leaf	dry	repellent	1	w
*Taraxacum officinale* F. H. Wigg. **	Cichoriaceae	ljekoviti maslačak	flower, root, whole plant, leaf	infusion, dandelion syrup, honey, compression, cream, salad, root juice	D: gastrointestinal problems; R: respiratory problems; U: urinary tract infection, detoxification; B: immune system; T: for weight loss; S: reducing inflammation, eczema, facial hydration; food	21	w
*Thymus serpyllum* L. **	Lamiaceae	majčina dušica, timijan	herb, leaf	spice, infusion	D: gastrointestinal problems; X: menstrual problems; N: neurological problems; food	6	c
*Tilia cordata* Mill. +	Tiliaceae	malolisna lipa	flower	infusion	A: fever; D: digestive problems, diarrhea; R: cold, cough	5	w
*Tilia* sp. *	Tiliaceae	lipa	leaf, flower	infusion, juice, honey	R: respiratory problems, cold; N: neurological problems; P: insomnia; B: immune system; food	18	w
*Trifolium repens* L. *	Fabaceae	bijela djetelina	flower	infusion	X: genital system	2	w
*Ulmus minor* Mill. *	Ulmaceae	poljski brijest	bark	ointment	S: dermatological problem	1	w
*Urtica dioica* L. **	Urticaceae	obična kopriva	leaf, whole plant, flower	raw, sauce, soup, macerate, juice, syrup, infusion, salad, shampoo	B: anemia, immune system booster; D: gastrointestinal problems; L: musculoskeletal; K: cardiovascular; U: urinary tract infection; N: neurological, U: detoxification; R: cold; S: hair loss, natural shampoo, allergies; against aphids, animal food, food	33	w
*Valeriana officinalis* L. **	Valerianaceae	ljekoviti odoljen	root	infusion	P: agitation, insomnia; T: hormone imbalance; X: menstrual problems	6	w
*Valerianella locusta* (L.) Laterr. *	Valerianaceae	obični matovilac	leaf	salad	food	1	c
*Verbascum* sp. *	Scrophulariaceae	divizma	flower	dry, boiled in milk	D: gastrointestinal problems	1	w
*Verbena officinalis* L. *	Verbenaceae	verbena	flower	honey	food	1	c
*Viola odorata* L. *	Violaceae	mirisava ljubica	leaf, flower	infusion	R: respiratory problems	2	w
*Viola tricolor* L. *	Violaceae	maćuhica	flower	fresh	food	1	w
*Viscum album* L. *	Santalaceae	bijela imela	leaf	infusion	K: cardiovascular	1	w
*Zea mays* L. *	Poaceae	kukuruz	corn silk	infusion	U: urinary tract infection	2	c
*Zingiber officinale* Roscoe +	Zingiberaceae	đumbir	root	infusion, fresh juice, spice	D: heartburn, nausea, flatulence; L: anti-inflammatory	9	p

Status: w—wild, c—cultivated, p—purchased plant; A, B, D, F, H, K, L, N, P, R, S, T, U, X: abbreviations for ailment category; FC—frequency of citation; * plants that were recorded in the Đurđevac area; + plants that were recorded in the Valpovo area; ** plants that were recorded in the Đurđevac and Valpovo areas.

**Table 2:** Informant consensus factor (Fic) of ailment category and the most frequently mentioned plants with the number of use reports (UR).

Ailment Category	Nur	Nt	Fic	The Most Frequently Mentioned Plants with UR
A—general and unspecified	10	8	0.22	*Mentha* x *piperita* 3
B—blood, blood-forming organs, and immune mechanisms	73	23	0.69	*Rubus plicatus* 11, *Urtica dioica* 7, *Allium sativum* 7
D—digestive	184	45	0.76	*Origanum vulgare* 25, *Mentha* x *piperita* 25
F—eye	11	2	0.9	*Chamomilla recutita 9*
H—ear	4	1	1	*Sempervivum tectorum* 4
K—cardiovascular	62	17	0.74	*Urtica dioica 19, Allium sativum 9*
L—musculoskeletal	18	10	0.47	*Aesculus hippocastanum 3, Armoracia rusticana 3, Brassica oleracea 3, Symphytum officinale 3*
N—neurological	45	13	0.73	*Chamomilla recutita 10*
P—psychological	31	9	0.73	*Chamomilla recutita 13*
R-–respiratory	158	26	0.84	*Chamomilla recutita 30, Salvia officinalis 20*
S—skin	122	23	0.82	*Calendula officinalis 23, Chamomilla recituta 19*
T—endocrine/metabolic and nutritional	27	8	0.73	*Cinnamomum verum 14, Apium graveolens 4*
U—urological	71	20	0.73	*Urtica dioica 10, Equisetum arvense 10*
X—female genitals	35	9	0.76	*Chamomilla recutita 9*

Nur—number of use reports, Nt—number of taxa.

**Table 3:** Mushrooms recorded in this study in the north-western part of Croatia.

Botanical Name	Local Name	Family	FC
*Agaricus campestris* L. 1753.	pećurka	Agaricaceae	11
*Agaricus macrosporus* (F.H. Møller and Jul. Schff.) Pilat)	kračun, velika pećurka	Agaricaceae	1
*Armillaria mellea* (Vahl) P. Kumm., 1871	puza, medenjača, kljukača	Physalacriaceae	2
*Boletus aereus* Bull. ex Fr., 1789.	hajdinski vrganj	Boletaceae	2
*Boletus edulis* Bull. ex Fr., 1782.	ljetni vrganj	Boletaceae	18
*Boletus pinophilus* Pilát and Dermek	borov vrganj	Boletaceae	2
*Cantharellus cibarius* Fr.	lisičarka	Cantharellaceae	5
*Flammulina velutipes* (Curtis) Singer	panjevčica	Physalacriaceae	5
*Hygrophorus eburneus* (Bull.) Fr.	bijela puževica	Hygrophoraceae	1
*Lactarius deliciosus* (L. ex Fr.) S.F. Gray	obična rujnica	Russulaceae	2
*Lactarius piperatus* (L.) Pers.	paprena mliječnica	Russulaceae	2
*Langermannia gigantea* (Batsch ex Pers.) Rostk.	golema puhara	Agaricaceae	1
*Leccinum aurantiacum* (Bull.) Gray 1821	turčin	Boletaceae	3
*Leccinum pseudoscabrum* (Kallenb.)	grabov djed	Boletaceae	3
*Macrolepiota procera* (Scop.) Singer	velika sunčanica	Agaricaceae	10
*Pleurotus ostreatus* (Jacq. ex Fr.) P. Kumm. 1871	bukovača	Polyporaceae	4
*Russula vesca* Fr.	jestiva krasnica	Russulaceae	1

FC—frequency of citation.

## Data Availability

The data presented in this study are available on request from the corresponding author. The data are not publicly available due to privacy reasons.

## Supplementary Materials

The following supporting information can be downloaded at: https://www.mdpi.com/article/10.3390/plants13111566/s1, Table S1: Interview with pre-planned questionnaires for local informants from Valpovo and Đurđevac area.
